# Language Learning Under Varied Conditions: Neural Indices of Speech Perception in Bilingual Turkish-German Children and in Monolingual Children With Developmental Language Disorder (DLD)

**DOI:** 10.3389/fnhum.2021.706926

**Published:** 2022-01-04

**Authors:** Tanja Rinker, Yan H. Yu, Monica Wagner, Valerie L. Shafer

**Affiliations:** ^1^Department of German as a Foreign and Second Language, Catholic University of Eichstätt-Ingolstadt, Eichstätt, Germany; ^2^Department of Communication Sciences and Disorders, St. John’s University, Queens, NY, United States; ^3^Ph.D. Program in Speech-Language-Hearing Sciences, The Graduate Center, The City University of New York, New York City, NY, United States

**Keywords:** bilingualism, developmental language disorder (DLD), electrophysiology, T-Complex ERP, auditory processing, speech processing

## Abstract

Lateral temporal measures of the auditory evoked potential (AEP) including the T-complex (positive Ta and negative Tb), as well as an earlier negative peak (Na) index maturation of auditory/speech processing. Previous studies have shown that these measures distinguish neural processing in children with typical language development (TD) from those with disorders and monolingual from bilingual children. In this study, bilingual children with Turkish as L1 and German as L2 were compared with monolingual German-speaking children with developmental language disorder (DLD) and monolingual German-speaking children with TD in order to disentangle effects of limited language input vs. reduced perceptual abilities in the processing of speech and non-speech stimuli. Sensory processing reflected by the T-complex (or from lateral temporal electrode sites) was compared in response to a German vowel and a sine-wave tone in the three groups of children, ages 5 through 6 years. Stimuli were presented while children watched a muted video. Auditory evoked potentials (AEPs) were time-locked to the vowels and tones. AEPs to the frequent (standard) stimuli within an oddball paradigm were analyzed at the left (T7) and right (T8) temporal electrode sites.The results revealed language status (monolingual, bilingual, and DLD), stimulus (vowel and tone), and language test measures (receptive and expressive) all influenced the T-complex amplitudes. Particularly, the peak amplitude of Ta was modulated by language status and stimulus type. Bilingual children had significantly more negative Ta responses than the monolingual children with TD for both vowels and tones while DLD children differed from TD children only for the vowel stimulus. The amplitude of the T-complex was overall more negative at the left than at the right site. The Na peak latency was longer for the bilingual group than that observed for the two monolingual groups. The Tb latency was shorter for DLD and bilingual groups than that for TD children in the vowel condition, but no such latency difference between DLD and bilingual children was found. We suggest that the attenuated T-complex for bilingual children indicates continued plasticity of the auditory cortex to allow for learning of novel, second-language speech sounds.

## Introduction

It has been well-documented in the literature that children are born with the ability to distinguish and categorize most speech sounds from any of the world’s languages. Within the first year of life, this ability narrows as the infant becomes attuned to the sound patterns of the ambient language(s) (e.g., Kuhl et al., 1992, 2008; Cheour et al., 1998). Neurobiological studies of speech and language have begun to reveal how intrinsic and environmental factors modulate the development of the child’s phonological system (e.g., Kuhl, 2010; Shafer et al., 2011b; Yu et al., 2019).

Both, bilingual development as well as disordered or delayed language development are two such cases that modulate the development of the phonological system. Hence, this study is concerned with investigating and comparing the phonological processing of three groups of children (monolingual, bilingual, and with language disorder) using a neural measure suited to analyzing the processing of fine-grained auditory detail (T-complex). Also, this study investigates a lesser-studied bilingual group of children, namely Turkish-German children living in Germany. For these children, Turkish is the L1 and German is the L2.

### Bilingual Phonological Development

Evidence suggests that experience with a second language early in life (under 5 years of age) allows for native or native-like discrimination and categorization of speech sounds from both languages (Flege et al., 1997; Bosch and Sebastián-Gallés, 2003; Hisagi et al., 2015). However, few studies have closely examined the development of L2 speech perception in the years before school entry. The studies that have addressed this question suggest that L2 speech perception and processing differ from monolingual children even after 2 years of exposure to the L2 (e.g., in daycare settings as in Rinker et al., 2010). In addition, considerable variability is observed for L2 phonological development in the years before school entry with variability evident as late as 4–6 years of age. A variety of factors, such as the input situation, language similarity, and the age of first exposure may account for variability in the time course of L2 phonological development, in addition to factors such as transfer or delay (Goldstein and McLeod, 2012; Core and Scarpelli, 2015).

To date, few studies of phonological development have focused on L2-learning of German by Turkish children (e.g., Ünsal and Fox, 2002; Albrecht, 2017; Fox-Boyer et al., 2020) although Turkish-German children constitute the largest bilingual group in Germany. Many studies of bilingual phonological development have focused on English as the L2, and/or on Spanish L2 learners (e.g., Spanish-English children: Fabiano-Smith and Goldstein, 2010; Shafer et al., 2011a, 2015; Yu et al., 2019; Spanish-German children: Kehoe, 2002).

The studies of Turkish-German learners by Albrecht and colleagues examined the development of phoneme productions longitudinally with the aim of analyzing typical and atypical processes in bilingual Turkish-German children raised in Germany (Albrecht, 2017; Fox-Boyer et al., 2020). They reported that the amount of input in the German language influenced the accuracy of German phoneme productions. For Turkish phoneme productions, the results were less clear, possibly because there was considerable variability in the amount of Turkish vs. German input at home. Clearly, additional studies are needed to further understand the relationship between language input and phonological development in this population.

### Developmental Language Disorder

The establishment of the phonological system is one of the essential bases for further language learning. Children identified as language impaired, i.e., having specific language impairment (SLI) or more recently called Developmental Language Disorder (DLD) often exhibit poor phonological development and skills, as well as poor word-learning and grammatical skills. For the remainder of the article, we will use the term DLD (Bishop et al., 2017). Poor phonological skills are seen as lower performance on speech discrimination and identification tasks (Sussman, 1993; Shafer et al., 2005; Datta et al., 2010) and phonological memory tasks (Briscoe et al., 2010; Claessen and Leitao, 2012). Children who show deviant patterns of processing speech in the first few months of life exhibit an increased risk for later language deficits (e.g., Friedrich et al., 2004; Weber et al., 2005; Cantiani et al., 2019).

Some researchers have claimed that deviant patterns of speech processing in children with DLD may be related to poor auditory processing skills (Tallal and Piercy, 1973; Tallal et al., 1993; Benasich and Tallal, 2002). Early disturbances in auditory and/or phonological processing appear to have persistent consequences for language development, particularly for reading. A significant proportion of children with DLD are diagnosed with dyslexia at school age (Catts et al., 2005; Boada and Pennington, 2006; Pennington and Bishop, 2009; Rispen and Baker, 2012). Even so, mixed findings have led to continued controversy about the relationship between DLD and auditory processing skills (Bishop et al., 2012; Moore et al., 2013). Clearly, more research is needed to fully understand how speech and non-speech auditory skills contribute to DLD (Tallal et al., 1993; Bishop and McArthur, 2004).

### Neural Measures of Speech Processing

Neural measures of speech processing appear to be particularly sensitive to differences in L2 phonological processing but also to impaired phonological processing. These measures can reveal differences in processing that are not observed at the behavioral level (e.g., Sebastián-Gallés et al., 2006; Hisagi et al., 2015).

Measures of speech encoding in the brain can also provide valuable information as outlined below. However, most studies of children with DLD and/or bilingual input have focused on speech discrimination (e.g., Cheour et al., 2002; Peltola et al., 2005; Rinker et al., 2010; Shafer et al., 2011b; Yu et al., 2019; DLD: Shafer et al., 2005; Datta et al., 2010; Kujala and Leminen, 2017). Measures of neural encoding include the T-complex auditory-evoked potentials (AEPs).

### The T-Complex

AEPs reflect mainly cortical-level brain activity that is time-locked and phase-locked to an auditory stimulus. The T-complex AEPs are recorded from electrodes overlying the lateral-temporal cortex and reflect obligatory sensory processing. T-complex measures in response to auditory stimuli, including speech and non-speech, have been shown to be sensitive to language abilities (Tonnquist-Uhlén, 1996; Shafer et al., 2011a) and language experience (Wagner et al., 2013; Rinker et al., 2017).

The T-complex measures were first examined in healthy adults by Wolpaw and Penry (1975). AEP responses from the temporal sites T3 and T4 (T7 and T8, respectively in the newer 10–10 notation) were initially elicited to a series of clicks in a time-window between 75 ms and 225 ms. These peaks were labeled Ta, a positive peak between 105 ms and 115 ms, and Tb, a negative peak between 150 ms and 160 ms. An earlier Na peak between 50 and 100 ms in adults, which might at least partially reflect the inversion of P1 at fronto-central sites, can also be observed at temporal sites (and was not measured in Wolpaw and Penry, 1975).

Dipole modeling indicates that neural generators of the T-complex can be traced to bilateral radial dipoles in the temporal lobe; this source orientation is consistent with secondary auditory cortex activity (e.g., Ponton et al., 2002).

A range of more recent studies extended the use of clicks to e.g., tones or speech. It was found that the Ta to tones and syllables was more prominent over the right than the left hemisphere both in younger children and teens, with syllables being even more prominent (Bishop et al., 2012) and a similar orientation towards the right for a range of speech stimuli (Shafer et al., 2011a). Only one study found that Na and Tb were more prominent on the left side (Mahajan and McArthur, 2013).

In summary, measures of auditory processes obtained from sites over the lateral cortex show promise for furthering our understanding of language development. However, to date, too few studies have been undertaken in children (or adults) to have a full understanding of the processes indexed by these measures and these studies have examined only a few different stimulus types (tones or speech).

### Maturation of the T-Complex

A few maturational studies of the T-complex peaks reveal a protracted time course of development from 3 months of age into the teenage years (e.g., Ponton et al., 2002; Bishop et al., 2012; Shafer et al., 2015). Specifically, Shafer et al. (2015) revealed in a developmental study from 3 months to 7 years of age that only Na is consistently present to a vowel stimulus in children under 4 years of age. The Ta and Tb peaks were clearly distinct from the child’s P100 response recorded at the vertex (Cz). The Ta peak emerges between 4 and 8 years of age. Tb was not easily identified in the children’s data, but it was found in adult data (Shafer et al., 2015). Studies of T-complex maturation across grade-school and into adulthood suggest that the Ta-peak amplitude first increases from approximately 7–11 years of age, and then decreases in amplitude up to adulthood (Albrecht et al., 2000; Tonnquist-Uhlén et al., 2003; Dunn and Bates, 2005; Mahajan and McArthur, 2013). The longitudinal study by Bishop et al. (2011) indicated stability in the T-complex measures from 9 to 11 years of age.

These findings, taken together, suggest that the generators underlying the Ta and Tb peak are highly immature before 4 years of age. Additionally, the generators may have a different orientation in infants and toddlers, leading to the absence of the peaks at the left (T7) and right (T8) temporal sites. The Na peak may partially reflect the opposite pole of the fronto-central P1 (P100; Shafer et al., 2015). P100 is the most prominent peak in young children’s AEP data, and thus, the inversion of the P100 peak, seen as a negativity at inferior sites, may partially overlap with other activity recorded at temporal sites that co-occurs in the same time frame (Shafer et al., 2015).

### The T-Complex to L2

To our knowledge, few studies have examined the T-complex in L2 learners. One study, focusing on 4- to 6-year-old children, observed differences related to input (Rinker et al., 2017). Monolingual and bilingual children from two different countries and language backgrounds participated: Spanish-English children from the US and Turkish-German children from Germany were compared to monolingual English-speaking and monolingual German-speaking children. In both experiments, neural responses to the vowel phoneme /ε/, which is found in both German and English, were examined. In the Turkish and Spanish languages, the German phoneme /ε/ and English phoneme /ε/ are perceived as a variant (allophone) of the Turkish /e/ and Spanish /e/, respectively. For native speakers of Turkish and Spanish, however, non-native German and English /ε/ will diverge from the prototypical phonetics of Turkish /e/ and Spanish /e/. The results revealed differences between the bilingual and the monolingual groups. Neural responses measured at the temporal sites were modulated by language experience, particularly in the Ta-range. Overall, Ta peaks were less well-formed and less positive in amplitude in many of the bilingual children. The authors suggested that more limited exposure to the L2 phonology resulted in less mature T-complex patterns (Rinker et al., 2017).

### The T-Complex in Children With Developmental Disorders

A few studies have examined the T-complex in children with developmental disorders and have found deviant or attenuated responses. Groen et al. (2008) found atypical lateralization of Ta amplitudes in children with Down’s Syndrome. Another study observed delays in the Tb latency in children with autism (Bruneau et al., 1999).

Only a small number of studies have examined the T-complex in children with DLD. Each of these studies observed differences in T-complex measures between children with DLD and TD. One study examined the general morphology of the waveform at temporal sites in the latency range from Na to Tb (Bishop et al., 2007). In another study, Ta amplitude was specifically observed to be attenuated in children with DLD (Shafer et al., 2011a). In this report, a variety of linguistic stimuli were investigated: vowels, real words, nonsense words, and the function word “the”. Specifically, children with DLD exhibited attenuated neural responses to a 250-ms vowel /ε/ at the right temporal site (T8). A third study showed a similar attenuation of Ta: Bishop et al. (2012) observed differences between children with TD and DLD for the Ta-peak at site T8 to a speech syllable (“bah”). In addition, they tested non-verbal stimuli (a 1,000-Hz tone). Results showed a significantly reduced Ta on the right (T8) for children with DLD compared to TD. Also, the study revealed that teens with DLD (around 14 years of age) had a reduction of Ta-amplitude compared to children with TD for both stimulus types.

### The T-Complex in Speech vs. Non-speech

Only one other study (than Bishop et al., 2012), to our knowledge, has directly compared T-complex measures to speech (vowel) and non-speech (1,000-Hz tone; Eulitz et al., 1995). As mentioned above, Bishop et al. (2012) found a similar deviant pattern for speech and non-speech stimuli in children with DLD. Eulitz et al. (1995) examined adults and observed greater positive amplitude over the left temporal site in the time range following the N1 for speech compared to non-speech stimuli; no stimulus differences were observed over the right temporal site (Eulitz et al., 1995). Their finding suggested a special role for the auditory cortex underlying the left temporal site in processing speech.

### Functional Significance of T-Complex Measures

It currently is unclear whether T-complex measures index acoustic-level processing abilities or linguistic-level processing or a combination of both. If the T-complex, in part, reflects language-specific experience, then differences should be observed for L2 learners of a language who have not yet fully acquired the L2 phonological categories (e.g., Rinker et al., 2010). In this case, L2 experience should have little or no effect on processing non-speech tones (except in relation to lexical tone processing). The alternative is that T-complex reflects auditory maturation more generally. In this case, differences between bilingual and monolingual children would originate from exposure to acoustic stimuli within the environment.

The finding that children with DLD show an attenuated T-complex to both speech and tones suggests poor auditory processing (e.g., Shafer et al., 2011a; Bishop et al., 2012). Children learning an L2, who do not have DLD, should not have poor auditory processing. Thus, if T-complex reflects general auditory processing skills, then bilingual and monolingual children should show no difference in T-complex measures for non-speech auditory information.

### The Present Study

The purpose of the current study was to test whether the different T-complex patterns observed for bilingual Turkish-German (L2 = German) and monolingual German children in Rinker et al. (2017) can be attributed to experience with the German vowel or to a more general modulation of auditory processing. To do this, we examined T-complex to both speech (German vowel /ε/) and non-speech (600-Hz tone). Also, we added children with DLD; this allowed us to further address whether differences in the T-complex observed for these children are related to general auditory processing or to a speech-related deficit. Testing all three groups and the two stimulus types facilitates our efforts to disentangle the effects of limited language input vs. purported perceptual abilities in the processing of verbal (German /ε/) and non-verbal stimuli. Differences between monolingual children and bilingual children can be attributed to limited input in the L2 (= effect of language experience). Bilingual children may show differences compared to monolingual learners because at the time of testing they had received less exposure to the L2.

We hypothesize that T-complex measures to both speech and non-speech are delayed or reduced for children with DLD (Shafer et al., 2011a; Bishop et al., 2012). We also predict that there is a correlation between children’s language skills, particularly phonological memory, and neural responses. For the bilingual children, we predict that the T-complex to non-speech stimuli will not differ from the monolingual TD children, as the processing may not be dependent on language experience. These bilingual children are the same as those in Rinker et al. (2017), and thus, we have already reported that their responses to the L2 German vowel were attenuated compared to the monolingual German group.

## Methods

Sixteen German (mean age: 64.9 months (SD 4.3), range: 59–72 months, seven females) and 12 Turkish-German children (mean age: 63.7 months (SD 7.7), range: 55–81 months, five females) were recruited from local daycare centers in the city of Ulm, Germany. Fourteen monolingual German children (mean age: 61.7 months (SD 5.6), range: 49–73 months, six females) diagnosed with DLD were recruited through the University Hospital, Ear-Nose-Throat-Clinic/Department of Pedaudiology database and clinic. All children had been diagnosed by a licensed speech-language pathologist prior to participation in the study. Written consent was obtained from all parents. The study was conducted in accordance with the Declaration of Helsinki and was approved by the Ethics Committee of the Ulm University.

### Materials

All children underwent extensive language testing. All children had non-verbal standard IQ scores above 85 (Raven, 2002) and normal hearing thresholds (<20 dB in the 250–4,000 Hz range). Children with DLD underwent additional audiological testing which is reported in a separate article (Rinker et al., 2014). As detailed in Table 1, groups did not differ significantly regarding age or IQ. Receptive and expressive German language skills were measured by the respective subtests of a German-language development test (Grimm and Schöler, 1991). In this standardized language development test, children have to repeat sentences with increasing complexity (expressive skills) or re-enact a sentence using wooden toys (receptive skills). In a passive lexical test of Turkish (part one of CITO, Arnheim, NL) Turkish–German children scored 39 out of 60 points (“satisfactory”; see Table 1). This test is computer-based and children have to click on the correct picture out of four pictures displayed.

**Table 1:** Participant characteristics, including means and standard deviations (in parenthesis) for IQ, age, and language scores.

	TD German	TD Turkish-German	Difference (TD German/TD Turkish-German)	DLD	Difference (TD German/DLD)	Difference (DLD/Turkish-German)
n	16	12		14
IQ (SD)	106.4 (12.3)	102.7 (13.6)	*p* = 0.466	104.4 (11.5)	*p* = 0.659	*p* = 0.735
Age in months (SD)	65 (4.3)	63.7 (7.6)	*p* = 0.607	61.7 (5.6)	*p* = 0.086	*p* = 0.441
Receptive language* (SD)	52.1 (7.6)	44.7 (9.8)	*p* = 0.036	43.9 (5.7)	*p* = 0.003	*p* = 0.794
Expressive Language* (SD)	54.4 (7.3)	39.2 (7.4)	*p*< 0.000	30.8 (2.3)	*p*< 0.001	*p*< 0.001
Pseudo-word repetition* (SD)	45.5 (7.4)	43.3 (7.9)	*p* = 0.463	31.9 (3.4)	*p*< 0.001	*p*< 0.001
Turkish lexical test		39 (5.8)				

Also provided are p-values of t-test comparisons between groups. *Receptive, expressive, and pseudo-word repetition skills are displayed with T-scores (a T-score between 40 and 60 is considered within the normal range). Turkish lexical test: Max. score: 60.

The three groups differed significantly on various language measures (see Table 1). Both bilingual children and children with DLD differed from the TD monolingual children in their receptive and expressive language measures (between 1.5 SD and 2 SD below the mean on at least the expressive or the receptive test). However, only DLD and TD children differed in their non-word repetition skills.

A language and developmental questionnaire (available from the authors upon request) was administered to all groups. The mean age of acquisition of German for the Turkish-German children was 28.8 months (SD 11.6) but the current language input was quite mixed with children being exposed to a primarily German environment for part of the day and to a mixed Turkish-German environment at home. Further language background data for the bilingual population can be found in Rinker et al. (2017). The monolingual German children (TD and DLD) were not exposed to a second language at home.

#### Stimuli and Procedures

The speech stimulus was the vowel [ε], created by the Semi-synthetic Speech Generation method (SSG, Alku et al., 1999), and was based on vocal tract models from sustained /ε/ produced by a German speaker (see Rinker et al., 2010 for details). The duration of the stimulus was 250 ms. A 5-ms rise and fall time was applied at the beginning and the end of the stimulus waveform using Hanning-windowing. Amplitude was maintained at the same level (fluctuating by less than 1.2 dB) for the remainder of the stimulus. The fundamental frequency was 115 Hz and F1 and F2 were 370 Hz and 1,965 Hz, respectively. The sounds were presented through headphones at 90 dB SPL. The ISI was 650 ms. We used an oddball design which elicits both the T-complex and the mismatch negativity (MMN). A total of 595 stimuli were presented. The non-speech stimulus was a 600-Hz tone with a 150-ms duration with 5-ms rise and fall time. The sound intensity, ISI, and number of stimuli were the same as in the speech stimuli.

As this was an oddball design to elicit the mismatch negativity (MMN), both, the standard stimuli /ε/ and the 600 Hz tone occurred with a probability of 0.85 and deviant /e/ with a probability of 0.15. The results of that study are reported elsewhere (Rinker et al., 2010, 2014). The stimulus duration differs for the speech and tones because these values were selected based on values used in developmental studies of speech (e.g., Datta et al., 2010) or tones (e.g., Shafer et al., 2000; Morr et al., 2002).

Children watched a cartoon movie with the sound off and were instructed to focus on the movie. As this experiment was part of a larger study including four experimental conditions (about 10 min each), the total testing time was about 1 h including breaks.

#### Recording and Processing of the Data

Similar processing parameters were used as for the published reports examining vowel discrimination and vowel perception in Turkish-German children (Rinker et al., 2010, 2017). The EEG was recorded from 39 electrodes using the BrainAmp amplifiers (Brain Products, Gilching, Germany). Eye movements were monitored with electro-oculogram (EOG) electrodes attached below and at the outer canthus of the left eye. The reference was to the left earlobe. Electrode impedance was kept below 10 kΩ. The EEG was sampled at a rate of 500 Hz (on-line band pass filter was at 0.1–70 Hz). The offline analysis was conducted using IGOR Pro 8. Data were filtered using a lowpass filter of 30 Hz (FIR filter, end of passband 30 Hz, start of reject band 35 Hz, down 40 dB at 40 Hz, and then a roll-off of approximately 30 dB per octave). Data were then segmented (-100–600 ms). Epochs with amplitudes exceeding ±70 μV) were excluded from further analysis. Artifact-free EEG segments were averaged. Averaged data were re-referenced to an average reference and then baseline-corrected (pre-stimulus baseline of 100 ms).

### Analysis

All peaks (and valleys) were first selected by an automatic algorithm using IGOR Pro8. This algorithm used the first derivative to identify peaks and the second derivative to identify the change in direction (that is positive-going vs. negative-going). Authors TR and MW then used the following protocol to identify which of these peaks/valleys were the Na, Ta, and Tb:

(a) Three time-windows were selected from the grand average data at T7 and T8 to constrain the selection of individual peaks. Na was defined as a negative peak occurring in the range of 50–120 ms, Ta as a positive peak in the range of 70–170 ms, and Tb as a negative peak between 120 and 220 ms.
(b) Na and Tb were chosen as the most negative-going valley (generated by the automatic algorithm) in the defined time range; Ta was the most positive-going peak (identified by the automatic algorithm) in the constrained time range. If two peaks/valleys were identical in amplitude in the time-window and more than 15 ms apart, then the earlier one was chosen (see rule c). Peaks/valleys needed to be in sequence: Na, Ta, Tb.
(c) Rules (c and d) were both used here. If two peaks/valleys were identified within a specified time-window and were separated by <15 ms and amplitude differences were <0.20 μV, then the peak/valley in the time-window that was more positive/negative would be selected, unless rule (d) overruled this decision.
(d) If it was unclear which of two valleys to select as Na within the Na time-window, or if there was only one prominent valley across the three time-windows, then the P1 peak at Fz was used to select and label Na. For example, if two negative valleys of comparable amplitudes (less than 0.2 μV) were both in the Na time-window, then the one closer in latency to P1 was selected. If there was only one prominent valley in the waveform that fell outside of the defined windows for Na and Tb, then it would be labeled as Na, rather than Tb, if its latency was within 20 ms of the P1.
(e) Additionally, VS rated the unclear cases.

**Figure 1:**
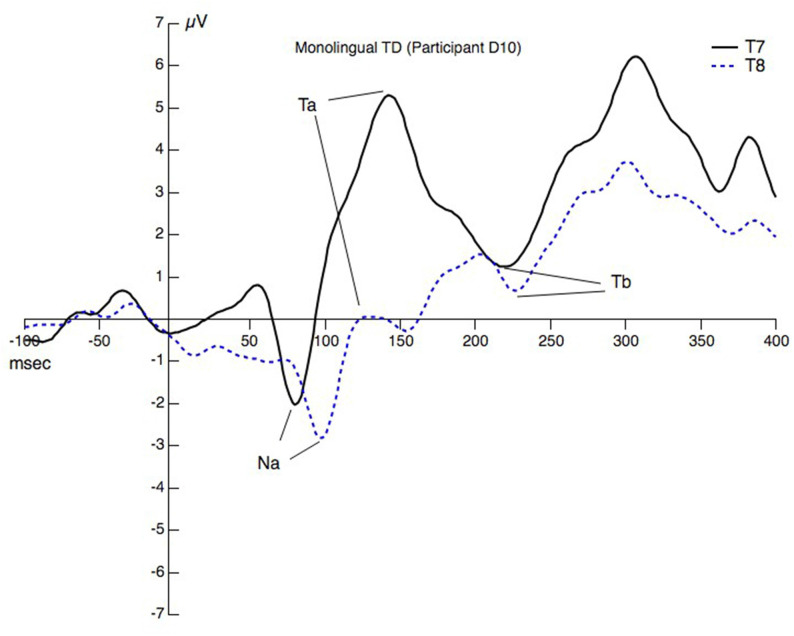
Peaks and valleys at T7 and T8 for one child. The peaks/valleys are assigned Na, Ta, and Tb and reflect some of the choices that needed to be made during the peak picking procedure.

Figure 1 displays the AEP at T7 and T8 with the selected peaks for one child (monolingual) to illustrate the procedure.

#### Statistical Analyses

To ensure that the Na-Ta-Tb responses are significantly distinguishable from noise, and distinguishable from each other, paired *t*-tests were performed on the amplitudes of Na, Ta, and Tb, and also on the differences between Na and Ta (Ta minus Na amplitude), and between Ta and Tb (Tb minus Ta amplitude). To examine the overall three-way main effects of language group, stimulus, and hemisphere, and their interactions, we used mixed-effects linear modeling and ran the analysis using the lme4 package in R (R Core Team, 2021). We then performed permutation ANOVAs to determine the main effect of language status (bilingual, monolingual, and DLD), stimulus effect (vowel vs. tone), and hemisphere (the left vs. right for the T-complex). Permutation analyses were used to control the multiple comparison problems that commonly occur in parametric statistical procedures including electrophysiological studies for the purpose of reducing Type I error (Maris and Oostenveld, 2007). Permutation Student’s *t*-test was used as the *post hoc* follow-up test. The analyses were performed in Rstudio using the RVAideMemoire package. In addition, correlations between language and T-complex measures were calculated using Pearson’s correlations with pairwise deletion of missing data.

## Results

**Figure 2:**
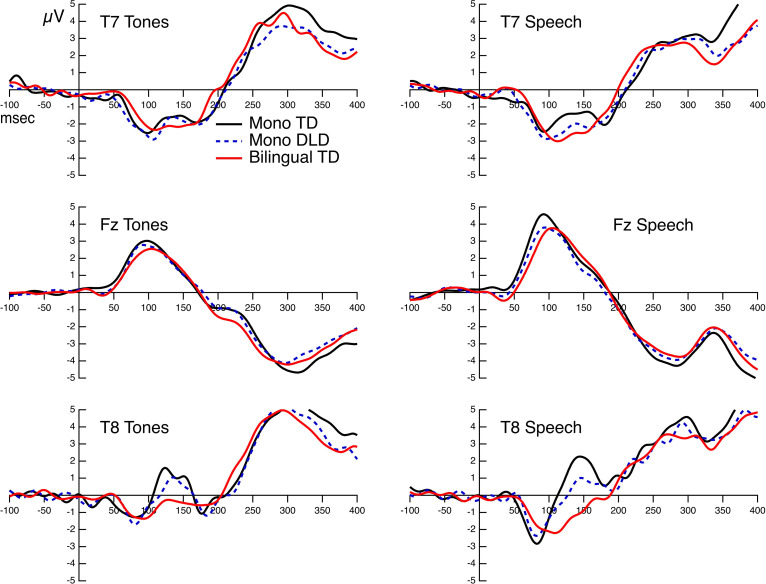
Grand mean AEPs at Fz, T7, and T8 for each stimulus. Responses for the three groups are overlaid on each graph.

The Grand Mean AEPs for the three groups and both stimuli at Fz, T7, and T8 are displayed in Figure 2. The morphology of the waveform at Fz roughly corresponded to the inversion of the Na peak. Na was the most easily identified peak for all subjects and is clearly identifiable for all groups in the Grand Mean. Tables s 2, 3 present the mean amplitudes and latencies for Na-Ta-Tb peaks.

**Table 2:** The peak amplitudes of the T-complex across participant groups and hemisphere sites.

	**T7**			**T8**		
Component peak	Na	Ta	Tb	Na	Ta	Tb
**Sine Tone (Mean amplitude)**	−3.05	−1.09	−2.68	−2.28	1.85	−1.63
SD	*1.80*	*2.01*	*2.76*	*1.47*	*2.48*	*2.22*
DLD German monolingual (Mean amplitude)	−3.56	−1.13	−2.93	−2.42	2.20	−1.81
SD	*1.79*	*2.31*	*2.45*	*1.52*	*1.99*	*1.82*
TD German monolingual (Mean amplitude)	−2.91	−0.68	−2.65	−2.46	2.84	−1.69
SD	*1.82*	*1.74*	*3.51*	*1.62*	*3.05*	*3.02*
Turkish-German bilingual (Mean amplitude)	−2.66	−1.53	−2.45	−1.92	0.32	−1.36
SD	*1.81*	*2.04*	*2.17*	*1.29*	*1.40*	*1.47*
**Vowel (Mean amplitude)**	−3.29	−0.86	−2.73	−3.14	1.65	−0.90
SD	*2.18*	*1.69*	*1.98*	*1.48*	*3.06*	*2.48*
DLD German monolingual (Mean amplitude)	−3.70	−1.26	−3.08	−2.89	1.17	−1.04
SD	*2.32*	*1.79*	*1.96*	*1.91*	*2.76*	*2.77*
TD German monolingual (Mean amplitude)	−3.05	−0.10	−2.47	−3.62	3.46	−0.37
SD	*2.12*	*1.66*	*2.38*	*1.29*	*3.42*	*2.77*
Turkish-German bilingual (Mean amplitude)	−3.09	−1.41	−2.60	−2.86	−0.05	−1.41
SD	*2.22*	*1.32*	*1.45*	*1.04*	*1.60*	*1.60*
**Grand Total (Mean amplitude)**	−3.17	−0.98	−2.70	−2.70	1.76	−1.30
SD	*1.98*	*1.86*	*2.41*	*1.53*	*2.75*	*2.36*

*Standard Deviations (SD) are displayed in the row below amplitude values (in μV) in italics*.

**Table 3:** The peak latencies of the T-complex across participant groups and hemisphere sites.

	**T7**			**T8**		
**Latency**	**Na**	**Ta**	**Tb**	**Na**	**Ta**	**Tb**
**Sine Tone (Mean amplitude)**	95.71	137.18	172.21	85.97	136.49	184.55
SD	*12.89*	*17.05*	*20.00*	*16.76*	*16.54*	*19.83*
DLD German monolingual (Mean amplitude)	95.99	135.32	168.33	82.92	137.31	185.54
SD	*14.27*	*16.17*	*15.29*	*17.83*	*15.32*	*17.15*
TD German monolingual (Mean amplitude)	97.20	139.36	177.27	83.93	132.21	191.00
SD	*11.81*	*20.31*	*22.34*	*16.88*	*15.05*	*17.97*
Turkish-German bilingual (Mean amplitude)	93.36	136.67	170.08	91.67	140.58	175.42
SD	*13.51*	*14.87*	*21.69*	*15.33*	*19.48*	*22.66*
**Vowel (Mean amplitude)**	97.38	130.77	165.23	88.05	139.74	175.50
SD	*13.77*	*20.17*	*18.25*	*16.16*	*18.44*	*23.24*
DLD German monolingual (Mean amplitude)	97.38	123.69	156.15	83.31	139.00	175.77
SD	*13.16*	*17.25*	*13.10*	*11.17*	*17.23*	*24.14*
TD German monolingual (Mean amplitude)	93.77	139.00	175.46	83.85	144.42	188.67
SD	*10.47*	*20.53*	*17.61*	*16.41*	*10.87*	*17.20*
Turkish-German bilingual (Mean amplitude)	101.64	129.11	163.56	98.64	134.56	157.56
SD	*17.52*	*21.34*	*19.66*	*17.10*	*27.04*	*18.02*
**Grand Total (Mean amplitude)**	96.52	134.15	168.95	86.99	138.00	180.39
SD	*13.27*	*18.74*	*19.40*	*16.40*	*17.41*	*21.79*

*Standard Deviation (SD) values are displayed In the row below latency values (in ms) in italics*.

### Presence of Na-Ta-Tb

Paired t-tests showed that Na, Tb, and the differences between Na-Tb, and Ta-Tb are all significant (*p*s < 0.001). However, the amplitude of Ta was not significantly different from zero (*p* = 0.21). Visual inspection revealed that the amplitude measures of T7 and T8 differed (more positive Ta at T8 than at T7). Therefore, *t*-tests were also performed on T7 and T8 separately; in this analysis, the Na, Ta, and Tb amplitudes are significantly different from zero and different from each other (Na vs. Ta; Ta vs. Tb) for both sites (*p*s < 0.001). The results from the permutation test were summarized in Table 4.

**Table 4:** Results from the permutation tests.

		**Latency**					**Amplitude**			
		*df*	Mean Sq	*F*	Pr(>F)		*df*	Mean Sq	*F*	Pr(>F)
Vowel*Tone	Na	1,150	135	0.55	0.45	Na	1,150	11.24	3.603	**0.06**
	Ta	1,145	99.06	0.3	0.58	Ta	1,145	0.0007	<0.001	0.99
	Tb	1,147	2,421	5.48	**0.02**	Tb	1,147	3.89	0.63	0.43
Tone										
Group	Na	2,75	57	0.23	0.8	Na	2,75	3.133	1.12	0.33
	Ta	2,75	57.5	0.2	0.82	Ta	2,75	18.8	2.72	**0.07**
	Tb	2,77	904	2.16	0.12	Tb	2,77	1.36	0.21	0.82
Vowel										
Group	Na	2,71	877.3	3.871	**0.02**	Na	2,71	0.9	0.255	0.77
	Ta	2,66	807	2.146	0.13	Ta	2,66	32.33	4.728	**0.01**
	Tb	2,66	2,752	7.132	**0.002**	Tb	2,66	2.617	0.4455	0.64
Tone										
T7:T8	Na	1,76	1,842	8.242	**0.007**	Na	1,76	11.6	4.303	**0.04**
	Ta	1,76	9.346	0.033	0.85	Ta	1,76	169.2	33.2	**<0.001**
	Tb	1,78	3,050	7.69	**0.008**	Tb	1,78	22.15	3.522	0.06
Vowel										
T7:T8	Na	1,71	1,608	7.133	**0.01**	Na	1,71	0.44	0.13	0.73
	Ta	1,67	1,386	3.706	0.058	Ta	1,67	109.5	18.1	**<0.001**
	Tb	1,67	1,819.5	4.181	**0.04**	Tb	1,67	57.5	11.5	**0.001**

*Note: p < 0.05 (in bold font)*.

### Mixed-Effects Modeling of Stimulus, Hemisphere, and Group

The results from Mixed-Effects Modeling showed that the only significant main effect is hemisphere for Na (*t* = 2.31, *p* = 0.02) and Tb (*t* = < 2.20, *p* = 0.03), with longer latencies at T7 than at T8 for Na, but shorter latencies at T7 than at T8 for Tb. No significant main effect or interaction was found for the latencies of Ta. Mixed-Effects Modeling on amplitudes revealed a significant main effect of hemisphere for Ta only (*t* = <4.5, *p* < 0.001) with high amplitudes (more positivity) at T8 than at T7. No other significant main effect or interaction was found.

#### Analysis of Amplitude Permutation Tests

*For Na*, a significant effect of the hemisphere was found. Larger Na (that is, more negative) amplitudes were observed at T7 than at T8 (*F*_1,76_ = 4.3, *p* = 0.04).

*For Ta*, a significant effect of the hemisphere was also found, but with smaller Ta (more negative) amplitudes at T7 than at T8 (*F*_1,76_ = 33.2, *p* < 0.001). For the vowel condition, the hemisphere effect was significant for Ta, with less positive/smaller Ta at T7 than at T8 (*F*_1,67_ = 18.1, *p* < 0.001). A significant effect of group was observed for Ta for the vowel condition (*F*_2,66_ = 4.7, *p* = 0.01), but only approached significance for the tone condition (*F*_2,75_ = 2.7, *p* = 0.07). *Post hoc* tests for the group effect in the vowel condition showed that the DLD group had less positive Ta amplitude than the TD group (*t* = −2.0, *p* = 0.04), and the bilingual group had less positive Ta amplitude than the monolingual TD group (*t* = 2.9, *p* = 0.002), but the DLD and the bilingual groups do not differ from each other (*t* = 1, *p* = 0.32). We chose to follow up on the tone difference, even though it did not reach significance to allow greater insight into the patterns in the subgroups. *Post hoc* tests for the tone condition showed that the DLD and TD monolingual groups do not differ from each other (*t* = −0.7, *p* = 0.49); in contrast, the bilingual group had less positive Ta amplitude than the DLD and TD monolingual group (*t* = 1.7, *p* = 0.05 for DLD vs. bilingual group; *t* = 2.339, *p* = 0.01 for TD monolingual vs. bilingual group).

For *Tb* in the vowel condition, the hemisphere effect was significant, with greater negativity at T7 than at T8 (*F*_1,67_ = 11.5, *p* < 0.001). For Tb in the tone condition, the hemisphere difference approached significance (*F*_1,78_ = 3.5, *p* = 0.06), similarly with more negative Tb at T7 than T8.

#### Analysis of Latency Permutation Tests

For *Na*, a significant effect of hemisphere was found for both the vowel and tone conditions (Vowel Na: *F*_1,71_ = 7.1, *p* = 0.01; Tone Na: *F*_1,76_ = 8.2, *p* < 0.01) with longer latencies at T7 than at T8 for Na for both stimulus conditions. A significant effect of group was observed only for the vowel condition (*F*_2,71_ = 3.8, *p* = 0.02). *Post hoc* tests revealed that for Na, the two monolingual groups do not differ (*t* = 0.4, *p* = 0.71), but that the bilingual group showed a longer Na peak latency than observed for the two monolingual groups (TD monolingual: bilingual: *t* = −2.5, *p* = 0.01; DLD: bilingual: *t* = −2.2, *p* = 0.03).

For *Ta*, an effect of hemisphere approached significance for Ta for the vowel condition with shorter latencies at T7 than at T8 (*F*_1,67_ = 3.7, *p* = 0.058).

For *Tb*, the permutation tests revealed that there was a significant difference of stimulus condition (*F*_1,147_ = 5.5, *p* = 0.02), with the latency of the vowels being longer than that of the tones (vowels = 178 ms, tones = 170 ms). A significant effect of hemisphere was found for both the vowel and tone conditions (Vowel Tb: *F*_1,67_ = 4.2, *p* = 0.04; Tone Tb: *F*_1,78_ = 7.7, *p* < 0.01) with shorter latencies at T7 than at T8 for both stimulus conditions. A significant effect of group was observed only for the vowel condition (*F*_2,67_ = 7.1, *p* < 0.01). *Post hoc* tests revealed that the DLD and the bilingual group do not differ (*t* = 0.9, *p* = 0.39); however, the bilingual group had a shorter Tb latency than the monolingual TD group (*t* = 3.7, *p* < 0.001), and the DLD group had a shorter Tb latency than the monolingual TD group (*t* = −2.8, *p* < 0.01).

#### Correlations Between Standardized Language Measures and the T-Complex Measures

Results from the Pearson correlation analyses for the vowel condition revealed a significant correlation between EG and Ta latency at T7 (*r* = 0.35, df = 22, *p* = 0.04), and the correlation between NWR and Ta latency at T7 was trending toward significance (*r* = 0.31, df = 33, *p* = 0.07). At the T8 site, for the vowel condition, a significant correlation was observed between RG and Na amplitude (*r* = 0.39, df = 32, *p* = 0.02); the negative correlation between EG and Na amplitude at T8 was trending toward significance (*r* = −0.31, df = 32, *p* = 0.07). However, after adjusting for multiple comparisons using Bonferroni correction (familywise alpha of *p* < 0.1, resulting in 0.1/36 = 0.0028), none of these correlations reached significance. Table 5 shows the correlation coefficients for all the comparisons. No significant correlations were observed between the T-complex and behavioral measures under the tone condition.

**Table 5:** Correlation between language measures and the amplitude and latency of the T-complex, respectively.

		**Tone**						**Vowel**					
		**T7**			**T8**			**T7**			**T8**		
		**Na**	**Ta**	**Tb**	**Na**	**Ta**	**Tb**	**Na**	**Ta**	**Tb**	**Na**	**Ta**	**Tb**
**Amplitude**	RG	0.01	0.00	0.08	−0.18	−0.12	−0.32	0.26	0.12	0.03	***−0.39***	−0.05	−0.28
	EG	−0.07	−0.06	−0.07	−0.13	−0.05	−0.29	0.09	0.16	0.10	**−0.31**	0.14	−0.07
	NWR	0.10	0.20	0.11	−0.05	−0.19	−0.19	0.20	0.25	0.26	−0.12	0.12	0.00
**Latency**	RG	0.14	0.24	−0.04	−0.08	−0.14	−0.05	0.00	0.22	0.03	−0.01	0.04	0.12
	EG	0.10	0.18	0.05	−0.08	−0.23	−0.01	−0.01	**0.35**	0.30	−0.03	0.09	0.20
	NWR	−0.09	−0.02	−0.02	0.03	−0.28	0.04	0.06	**0.31**	0.15	0.21	0.17	−0.12

*Note: *p* < 0.05 (in bold font)*. RG, receptive grammar; EG, expressive grammar; and NWR, nonword repetition.

**Figure 3:**
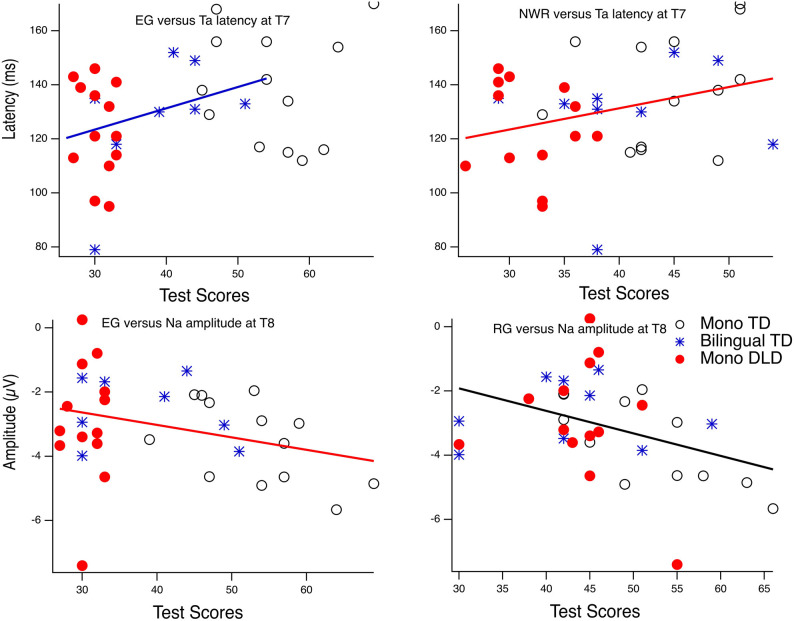
Correlationsbetween language tests and amplitude and latency measures in responseto the vowel stimuli. EG, expressive grammar; RG, receptive grammar;NWR, non-word repetition.

Figure 3 shows the regression line fit between the significant test score and T-complex measure, but also illustrates group membership for each participant.

## Discussion

This study explored the left and right temporal AEP measures Na, Ta, and Tb to the German vowel phoneme /ε/ and to a 600-Hz sine tone in monolingual and bilingual children with typical language development, as well as in children with DLD.

We found that both the children with DLD and those with bilingual input showed a less positive Ta amplitude for the vowel condition compared to the monolingual TD group. In contrast, for the tone condition, only the bilingual children showed a less positive Ta compared to the monolingual TD group. The children with DLD seemed to have a somewhat intermediate Ta response to the tones since their response did not differ from either the TD monolingual or the bilingual group. With respect to the latency measures, there were group effects at both Na and Tb, but only for the vowel condition. For Na, the bilingual group had longer latencies, while the monolingual groups (TD and DLD) did not differ. With respect to Tb, the DLD and the bilingual group did not differ and, in fact, the bilingual and DLD groups had shorter Tb latencies than the monolingual TD group.

We also observed effects of the hemisphere and of stimulus that did not interact with the group. The Na was generally more negative for vowels than for tones. The hemisphere effect was also significant in the mixed modeling approach used to examine three-way interactions between hemisphere, group, and stimulus. The children generally showed more negative amplitudes at the left than at the right sites for both tones and vowels, consistent with reports from previous studies (Shafer et al., 2011a, 2015; Bishop et al., 2012).

The Na and Ta peak amplitudes and latency measures were only weakly associated with some of the language measures. The left Ta latency was weakly correlated with expressive language skills (EG and NWR) and the right Na amplitude was negatively correlated with receptive language skills. However, after correcting for multiple tests, these relationships were not significant. Below, we discuss this pattern of findings in greater detail.

### The T-Complex Amplitude in Relation to L2 Input

We hypothesized that the T-complex amplitude and latency would be modulated by language experience. Specifically, an increasingly prominent Ta peak would reflect more phonological experience. This hypothesis led to the prediction that bilingual children would show an attenuated T-complex response to vowels, but not to tones. This hypothesis does not lead to a clear prediction for the monolingual children with DLD because they receive considerable input in their native language; however, it is possible that this input is less complex (to adjust to the child’s language level) and that the slower rate of learning (leading to a smaller vocabulary) is equivalent to less experience with the first language phonology. The finding of attenuated Ta to the vowel for the bilingual children compared to the TD monolingual group is consistent with this prediction; however, our finding of an attenuated Ta amplitude for tones between the monolingual TD group and the bilingual group does not unequivocally support this hypothesis. It is possible, however, that the pattern observed for the tone stimuli reflects some more general effect triggered by L2 experience. Below, we offer an explanation, after briefly discussing the pattern observed for children with DLD.

### Developmental Aspects of Temporal Site Responses

Both the children with DLD and the bilingual children showed a clear difference in the Ta amplitude to the vowels compared to the monolingual children with TD. In contrast, only the bilingual group showed a difference from the monolingual controls for the tone stimuli, but primarily in latency measures. As stated above, this pattern of findings only partially supports our hypothesis that language experience modulates the T-complex. Specifically, the bilingual group should have shown comparable T-complex responses to the monolingual TD group for the tones.

Unlike clinical populations who show delays in L1 acquisition, L2 learners (most of whom do not have a DLD) can be assumed to be competent in acquiring language. There is no reason to suspect that L2 child learners are poor phonological processors; however, their development of L1 and L2 speech perception and processing may still be delayed or different because they have less experience with each language or because they have experience of a different nature. A Turkish monolingual control group could have helped resolve the issue of whether the attenuated T-complex effects observed for the bilingual group are due to a different language background (Turkish) or due to specific limitations of the linguistic environment. Testing a German-Turkish bilingual group who were learning Turkish as an L2 could also address this question.

The T-complex measures in the bilingual children revealed immature patterns of sensory processing to acoustic stimuli. Specifically, the less positive Ta resembled the response in younger monolingual children (Shafer et al., 2015). Rinker et al. (2017) also included a Spanish-English bilingual group, who showed less positive Ta responses compared to English monolingual controls and these controls are the 4–5 years old children in Shafer et al. (2015). Thus, the pattern observed for bilingual children to a non-native vowel is replicated in two different language groups. Note that in the current study the Turkish and German data were filtered less strictly than in Rinker et al. (2017) to ensure that the Ta peak would not be suppressed. This current analysis showed the same general pattern of results as in Rinker et al. (2017). Specifically, less positive T-complex in the Turkish-German bilinguals compared to the German monolinguals. The novel finding in the current study, that Ta attenuation is also observed to simple tone stimuli in bilingual children will require replication. If replicated, the difference in T-complex measures in the bilingual group might reflect an immature pattern of sensory processing. Alternatively, the difference may be related to increased brain activation to auditory input in some neural populations that sums up at the scalp as less positive responses. Next, we discuss these considerations.

The introduction of an L2 early in life (perhaps before 5 years of age) may lead to maintaining a higher level of plasticity into the grade-school years. In other words, introduction of an L2 during this early time period leads to extending a sensitive period for learning the language-specific speech patterns (Werker and Hensch, 2015).

Previous studies have demonstrated declines in both perception and production of the fine-grained phonetic detail of an L2 with increasing age of acquisition (Flege et al., 1997; Hisagi et al., 2015). Studies of language ability and critical periods (which often have focused on grammar) have suggested a sensitive period for language input (we prefer “sensitive” to “critical”), sometime before adulthood, with some, but not all favoring the age of 5 years (Johnson and Newport, 1989; Birdsong and Molis, 2001; Hartshorne et al., 2018).

Our data provide important insight by revealing that the nature of the brain response to L2 speech sounds may extend to non-speech. This finding suggests a modulatory effect on auditory cortical responses, triggered by bilingual input and experience. More specifically, bilingual input may modulate neural responses to non-speech stimuli; languages differ in phonotactic patterns, as well as phoneme inventory, each constituting different patterns of sound energy concentration (Intartaglia et al., 2017). Thus, the input from phonological patterns from two languages might modulate the maturation of cortical areas which more generally support auditory processing. It will be important to examine whether this bilingual difference in the T-complex is maintained into the adult years. An increased negativity of the T-complex for native-Polish adults (late learners of English) compared to native-English adults to a Polish phonotactic pattern within the context of a challenging task demonstrated that linguistic-level processing was reflected within this sensory processing component (Wagner et al., 2013). Whereas this result is at least consistent with the pattern found for these children, more studies with both child and adult bilinguals are necessary to confirm these patterns.

Few studies have examined how early learning modulates the T-complex. An interesting study that focused on musical experience and absolute pitch (AP) perception, rather than speech, observed differences for these temporal measures in relation to these factors (Matsuda et al., 2019). Specifically, adult musicians (who all had childhood experience) compared to non-musicians showed greater asymmetry of the left vs. right T-complex, with the right showing more positive responses. In addition, those with AP showed increased negativity of the Na and Tb at the right site. The patterns of development across the childhood years, however, cannot be determined from this study, since the focus was on adults. Thus, it is currently unclear whether the increased negativity of the responses observed for the bilingual compared to monolingual children in our study would also be observed in older groups. Even so, these studies taken together demonstrate that both speech and non-speech auditory experience modulate T-complex responses.

The children with DLD further support our hypothesis that language experience can explain the pattern observed in our study. Specifically, the T-complex responses for a subset of the children with DLD to tones were very similar to children with TD. In contrast, an attenuated T-complex response in a child with bilingual input may indicate that the auditory cortex is plastic and allows modification. Future studies will be needed to test these hypotheses. In particular, it will be important to examine the T-complex in relation to the age of acquisition of the L2 and/or to follow children with bilingual exposure longitudinally to learn whether or not the T-complex for bilingual and monolingual children at older ages follow a similar trajectory.

### The T-Complex in Relation to Developmental Language Disorder

We had predicted that the children with DLD would show poor T-complex measures to both vowels and tones, indicating poor auditory processing. However, we only saw this pattern for the vowel condition and not the tone condition, suggesting a greater deficit for speech processing. Interestingly, a study by Helenius and colleagues found abnormal left temporal lobe responses in children with DLD in a “word” form repetition design and attributed this to a working memory deficit (Helenius et al., 2014). It will be of particular interest to further examine how the T-complex relates to a range of working memory measures.

There was no significant difference in the T-complex to the tones for children with DLD compared to the two other groups. The figures show almost identical patterns for the children with TD and DLD for the Na-Ta-Tb AEPs at both left and right sites.

The (weak) correlation of Na to the vowels with the EG and RG language scores appears to be driven by a subset of the children with DLD showing attenuated Na (see Figure 3). Other studies of children with DLD also have shown poor T-complex to speech (Shafer et al., 2011a; Bishop et al., 2012). Future studies will be needed with a larger N, but which also provide a more fine-grained measure of the language deficits of children with DLD to fully understand the association between T-complex measures and language behaviors in children with DLD. We discuss this further below in relation to various co-morbidities, such as dyslexia.

### Heterogeneity of DLD

As mentioned above, one potential contributing factor to our lack of finding of a difference between the children with DLD and those with TD for the tone condition is variability, which might be due to the heterogeneous nature of DLD (Shafer et al., 2011a; Bishop et al., 2016). It is possible that only a small proportion of the children with DLD in the current study had phonological processing deficits. Children with comorbid DLD and dyslexia (~30–40% of children with DLD), compared to those with DLD alone, were found to have phonological processing deficits (McArthur and Bishop, 2004; Catts et al., 2005; Boada and Pennington, 2006; Bishop et al., 2009; Rispen and Baker, 2012). These deficits might be caused by impaired processing of spectral (McArthur and Bishop, 2005; Ceponiene et al., 2009) and/or temporal acoustic characteristics (Tallal and Piercy, 1973; Benasich and Tallal, 2002). The children in the current study had not yet learned to read (children in Germany enter school at 6 years of age). Thus, it is unknown which of these children might have dyslexia (diagnosed at a later age). It will be important in a future study to examine whether poor T-complex measures are specifically associated with dyslexia.

### Limitations

The small samples of the current study and the absence of large effects warrant caution when interpreting the results. However, the three groups were clearly differentiated by the behavioral language measures and by input (bilingual vs. monolingual context). Thus, we can infer that it is unlikely that a large effect will be observed in a future study comparing children with TD and DLD, except in the case that heterogeneity is narrowed, as we suggest above, by examining reading skills.

With respect to the bilingual children, a monolingual Turkish control group would have helped to disentangle the effects of Turkish vs. German. This was not possible due to practical constraints. In addition, the observed effects need to be followed up with children growing up with other language combinations (such as English-Spanish or Italian-German).

The correlation between the T-complex measures and the different language scores were, at most, fairly weak and did not reach significance after correcting for the number of comparisons. In addition, the N for each group was too small to justify examining the relationship between these measures and each language group. A future study with more participants in each group will be necessary to determine whether language skill has a linear relationship to the T-complex measures.

Due to theoretical considerations guiding the original MMN-study (Rinker et al., 2010, 2014) the vowel and sine tone stimuli did not have the same duration. Future studies should compare processing for vowels and tones of the same duration.

A further limitation is that input patterns in German and Turkish were calculated from a parent questionnaire, thus potentially resulting in under- or overestimations of the input. Future studies would be better informed with a more direct measure of input, such as using LENA (García-Sierra et al., 2016) however, LENA has not yet been normed for use with typical heritage languages in Germany, such as Turkish.

## Conclusion

This study is the first to perform a three-way comparison of neural responses (T-complex) to speech and non-speech stimuli in monolingual children with TD, bilingual children with TD, and monolingual children with DLD. The main finding was that the T-complex measures to both speech and tones were modulated by language input and language processing abilities. As all children between 5 and 6 years of age are still showing a developing T-complex and heterogeneity among this age group is likely to be high, further studies are needed to elucidate the role between input, maturation, and disorder.

## Data Availability Statement

Publicly available datasets were analyzed in this study. Data is available here: https://dataverse.harvard.edu/dataverse/T-complex_mono_biling.

## Ethics Statement

The studies involving human participants were reviewed and approved by University of Ulm, Ethics Committee. Written informed consent to participate in this study was provided by the participants’ legal guardian/next of kin.

## Author Contributions

VS and TR conceived the research. TR carried out the research. MW and TR conducted preliminary analysis of the data. YY conducted initial data processing. YY and VS statistically analyzed data. All authors interpreted the results, wrote, and reviewed the manuscript and approved the submitted version. All authors contributed to the article and approved the submitted version.

## Conflict of Interest

The authors declare that the research was conducted in the absence of any commercial or financial relationships that could be construed as a potential conflict of interest.

## Publisher’s Note

All claims expressed in this article are solely those of the authors and do not necessarily represent those of their affiliated organizations, or those of the publisher, the editors and the reviewers. Any product that may be evaluated in this article, or claim that may be made by its manufacturer, is not guaranteed or endorsed by the publisher.
